# The mental health of Asian American adolescents and young adults amid the rise of anti-Asian racism

**DOI:** 10.3389/fpubh.2022.958517

**Published:** 2023-01-13

**Authors:** James Huynh, Jessie Chien, Amy T. Nguyen, Delanie Honda, Emily EunYoung Cho, Maliya Xiong, Tran T. Doan, Thoai D. Ngo

**Affiliations:** ^1^Department of Community Health Sciences, University of California, Los Angeles Fielding School of Public Health, Los Angeles, CA, United States; ^2^Darkness to Light, North Charleston, SC, United States; ^3^iMentor, New York, NY, United States; ^4^Population Council, New York, NY, United States; ^5^Wisconsin United Coalition of Mutual Assistance Associations, Wausau, WI, United States; ^6^Department of Pediatrics, Division of General Academic Pediatrics, University of Pittsburgh, School of Medicine, Pittsburgh, PA, United States

**Keywords:** mental health, Asian Americans, adolescents, young adults, anti-Asian racism, harassment, depression

## Abstract

**Objectives:**

We describe the perceptions and experiences of anti-Asian racism and violence and depression severity prior to and during the COVID-19 pandemic among a sample of Asian American (AA) adolescents and young adults.

**Methods:**

We used data from the Young Asian American Health Survey (YAAHS), an online-recruited sample of AA adolescents (ages 13–17) and young adults (ages 18–29 years) conducted during May 2021 to March 2022. We presented descriptive statistics examining the univariate distribution and bivariate relationships of depression severity, sociodemographic characteristics, and experiences and perceptions of anti-Asian violence.

**Results:**

Our sample (*n* = 176) comprised AA adolescents and young adults from 17 Asian ethnicities. A quarter said that the frequency and/or severity of their personal experiences of anti-Asian harassment had increased since the pandemic started. 76% indicated feeling less safe now than before the pandemic. Two-thirds reported that their depressive symptoms have increased since the pandemic started. Participants who reported feeling less safe now than before the pandemic were more likely to report increased personal experiences with anti-Asian harassment and increased depression severity since the pandemic started than those who reported feeling as safe or safer before the pandemic (*p* < 0.01 for both).

**Discussion:**

Findings illustrate AA adolescent and young adults are experiencing multiple health and social crises stemming from increased anti-Asian racism during the COVID-19 pandemic. We urge policymakers to strengthen data systems that connect racial discrimination and mental health and to institute prevention measures and anti-racist mental health services that are age- and culturally-appropriate for AA adolescent and young adults.

## 1. Introduction

Mental health conditions are increasing worldwide and are shaped by a variety of social factors, and racial discrimination in particular has been found to significantly impact risk for depressive symptoms among racially minoritized groups (1, 2). While the threat of the COVID-19 virus and pandemic effects are real for everyone living in the U.S., Asian Americans (AA) bear the additional burden of elevated anti-Asian sentiments and attacks (3–7). Such experiences and perceptions of heightened racial discrimination may act as a chronic social stressor that exacerbates adverse mental health among Asian Americans (8–10) and contribute to the existing rise in mental health struggles among Asian American adolescents and young adults.

AA adolescents and young adults are the fastest growing racial and ethnic population segment in the U.S. (11). Adolescence is a stage of rapid physical, social, and brain development; thus, AA adolescents and young adults may be especially vulnerable to increased depressive symptoms due to their exposure to multiple stressors associated with the COVID-19 pandemic, such as increased social isolation, family financial strain, and increased social media use. They also feared for their own safety as they became direct targets of anti-Asian hate crimes (12, 13).

Additionally, the health and well-being of adolescents (age 10–17) are distinctive from young adults (age 18–29), and therefore, the impacts of these stressors may vary between these two age groups. Despite the fragmented incidence data and being labeled as a “model-minority”, AA adolescents and young adults are still shown to be equally as vulnerable to developing mental health disorders as other racial/ethnic groups (14). Among adolescents aged 12–17 years, ~18.6% of non-Hispanic Asians have reported ever experiencing a major depressive episode, compared to 23.4% of non-Hispanic White/Caucasians, based on nationally representative data from the 2019 to 2020 National Survey on Drug Use and Health (NSDUH) (15). Among Asians from this dataset, about 24.5% Filipino, 20.3% Indian, 18.8% Vietnamese, 18.1% Japanese, 18.0% Korean, and 13.2% Chinese have ever experienced a major depressive episode (15). Across all Asian American adult age groups, young adults aged 18–25 years, comprised the highest group ever experiencing a major depressive episode, according to the 2019–2020 NSDUH.

AA adolescents and young adults are in a unique rapid developmental period, which makes them especially vulnerable to racial discrimination, heightening the anti-Asian climate during the pandemic. These adolescents and young adults are experiencing increased identity exploration and formation that is strongly tied to their racial/ethnic background and how their racial/ethnic identities are perceived by others (16, 17).

For example, one study found that Chinese American young adolescents who faced fewer developmental difficulties were associated with parents who socialized their children about maintaining their cultural heritage (18). These effects were not observed among older adolescents. However, the study found that both young and older adolescents with more developmental difficulties were associated with parental messages about concealing one's racial/ethnic identity during COVID-19 (18). Despite the growing recognition of the rise of anti-Asian racism in the era of COVID-19 in the national dialogue, there remains a data gap on the consequences of this increased racial discrimination on AA adolescents and young adults' mental health and well-being in the U.S. Without robust data, it is not possible to understand the breadth and depth of the intertwined effects of racism, violence, and mental health outcomes that can facilitate the design of effective and age-/culturally-appropriate public health policies and interventions to support this population group.

This climate of racial discrimination may be attributed to Orientalism and Sinophobia. Coined by Edward Said, orientalism is a framework to understand the colonial relationship between the West and the East. The East and its people, including Asia and the Pacific, are viewed through the Western gaze as subservient, savage, and in need of civilizing (read: conquest, colonizing) by the strong, sophisticated West (19). Contemporary anti-Asian racism during the COVID-19 pandemic has manifested in ways that build on this historical Orientalist context, specifically fomenting Sinophobia (i.e., anti-Chinese sentiments). In particular, those racialized as Asian in the U.S. have been flattened into a monolithic group and all assumed to be Chinese, and subsequently blamed for geopolitical tensions between the U.S. and China. As a result, those racialized as Asian in the U.S. are viewed as vectors of disease that could harm the U.S. body politic (4, 13, 20, 21).

The socio-ecological framework can be used to understand the multi-level structure and impacts of Orientalism. McLeroy et al. ecological model for health promotion posits that nested environmental levels (e.g., macro-, community-, interpersonal, and individual-level) affect individual behavior and health outcomes and vice-versa (i.e., individual behaviors may impact higher-scale levels) (22). As a macro-level ideological geopolitical climate, Orientalism structures the psychosocial and economic pathways that may impact the health of AAs. At the policy level, disinvestment in and gentrification of Chinatowns and other Asian ethnic enclaves throughout the U.S. reveal the structural violence in working-class communities, placing them at increased risk of poorer health outcomes (6, 23, 24). At the community level, this simultaneous disinvestment and gentrification occur because Chinatowns and other Asian ethnic enclaves represent sites where the working-class AAs are diseased (a perception even more pervasive because of COVID-19 first being discovered in Wuhan, China) and dirty (25), and therefore in need of sanitizing to fit an upper-middle class White aesthetic (26). At the interpersonal level, AAs, especially women, transgender, and gender non-conforming people, have been subject to racial discrimination, public harassment, and physical and psychological violence in places such as sidewalks, subways, and social media (3, 27, 28). These pathways can all influence AAs' mental health by exposing them to direct harm, chronic stress, feelings of hypervigilance and by undermining their sense of safety and belonging. Moreover, for AA adolescents and young adults, healthy racial and ethnic identity formation processes can be disrupted (16, 17).

In this article, our objective is to conduct a rapid assessment survey describing the perceptions and experiences of anti-Asian racism and depression severity prior to and during the COVID-19 pandemic among AA adolescents and young adults. We hypothesize that our online-recruited U.S. sample of AA adolescents and young adults are experiencing decreased levels of personal safety and increased levels of depression symptoms associated with either personal experiences or perceptions of a surge in anti-Asian sentiments, hate crimes, and violence during the on-going pandemic. Findings from this survey can help illuminate opportunities for health policymakers to invest in infrastructures that effectively improve the mental health statuses of AA young people impacted by recent anti-Asian hate during the COVID-19 pandemic.

## 2. Methods

### 2.1. Study design and sample

This study used data from the Young Asian American Health Survey (YAAHS) (29), an anonymous, self-administered, online cross-sectional survey for Asian American adolescents (aged 13–17 years) and young adults (aged 18–29 years) living in the United States. The YAAHS questionnaire was developed by the authors of this report. We adopted questions about personal experiences and perceptions of violence/harassment from the Pew Research Center's American Trends Panel survey, included the validated Patient Health Questionnaire-9 (PHQ-9) instrument widely used in the general population to assess depression status, and incorporated a few open response questions about people's experiences with and perceptions of anti-Asian violence/harassment. Before dissemination, we piloted our survey with a small group of Asian/Asian American adolescents and young adults (*N* = 27) to ensure our questionnaire was feasible, appropriate for a broad range of literacy levels, and relevant. We solicited volunteers for our pilot test *via* emails to our community partners' listservs and our social networks and ended pilot testing after 1 week. Based on feedback from our pilot participants on the readability of our survey, we distributed our survey in English only. We recruited study participants through direct contact *via* e-mail and postings on social media sites such as Facebook, Instagram, and Twitter. Population Council's IRB reviewed and approved this study (Protocol #975) for conducting human subjects research. The survey and datasets generated for this study can be found in the study's page on the Harvard Dataverse Repository (29).

We focused specifically on people who are racialized as Asian and Asian American, while excluding individuals who identify as Pacific Islanders. While Asians and Pacific Islanders are often grouped together, this obscures the distinct political, social, and economic histories of two heterogeneous groups. Pacific Islanders including Native Hawaiians, Samoans, Chamorros (indigenous people of Guam) have actively resisted being lumped with Asian and Asian Americans because of their experiences with U.S. settler colonialism and indigeneity (30–32). Since our study specifically seeks to better understand anti-Asian violence in the context of the COVID-19 pandemic during which more visible Orientalism and anti-Chinese geopolitics were observed, we restricted our scope to those identifying as Asian Americans. In doing so, we sought to resist the problematic grouping of Asians with Pacific Islanders and be able to highlight the experiences and impact of racism on Asian Americans to direct appropriate actions.

Survey responses were collected through SurveyMonkey from May 2021 to March 2022. Our eligibility criteria for being included in the study sample was self-identifying as Asian/Asian American, living in the US during the pandemic, and being between the age of 13–29. Among the 323 people who initiated a survey response, 306 respondents met our eligibility criteria. However, 130 (43%) of the eligible respondents were missing information on all key exposure variables; most of these missing cases were from unfinished questionnaires (i.e., respondents stopping shortly after initiating a survey response and thus not completing the rest of survey). Respondents who had incomplete data were not significantly different on race, ethnicity, or depression severity compared to those who had complete data. Therefore, we excluded respondents with incomplete data in our analysis, making our final analytical sample 176 participants.

### 2.2. Key measures

The survey asked close- and open-ended questions regarding participants' personal experiences and perceptions of anti-Asian violence before the pandemic and during the pandemic when there was a rise in anti-Asian violence, depressive symptoms, sociodemographic characteristics, and coping mechanisms under the current climate. There were also open-ended questions to elicit qualitative explanations for some of these items.

#### 2.2.1. Anti-Asian violence

##### 2.2.1.1. Experiences of anti-Asian violence

Participants were asked whether they experienced the following types of harassment and/or violence in-person or online before the pandemic (yes/no): offensive name-calling, purposeful embarrassment, physical threats, vandalism or destruction of personal property, and physical attacks. Participants then indicated if and how their personal experiences with each type of harassment had increased since the pandemic begun: yes, in frequency; yes, in severity; yes, in both frequency and severity; no; or not applicable. For analyses, we grouped all “yes” responses into one category and then created aggregate measures of whether they experienced any type of in-person and/or online harassment before the pandemic (yes vs. no) and whether their experiences of harassment had increased since the pandemic (yes vs. no. vs. never experienced harassment).

##### 2.2.1.2. Perceptions of safety and of anti-Asian violence

Participants were asked if they felt safe in their current neighborhood (safe vs. not safe) and to rate how safe they felt in their city/town now compared to how they felt before the pandemic (dichotomized as feeling less safe vs. feeling the same/more safe now than before the pandemic). Participants then reported if they had avoided public spaces due to fear of being a target of anti-Asian violence since the pandemic started (yes/no). Participants also rated their perceptions of changes in level of anti-Asian violence since the pandemic started; responses were dichotomized as a little worse/much worse vs. the same/little better/much better than before the start of the pandemic. Additionally, we asked participants to provide responses to open-ended questions on: (1) how they felt when they saw or heard about events related to hate crimes and racism toward Asian individuals and communities since the start of the pandemic; (2) how the level of anti-Asian violence has changed since the start of the pandemic; and (3) specific changes in their movements or behaviors in public spaces since the start of the pandemic due to fear of being a target of anti-Asian violence.

#### 2.2.2. Mental health outcomes

*Recent (i.e., in the past two weeks) depression severity* was measured using the 9-item Patient Health Questionnaire (PHQ-9), a commonly distributed questionnaire and diagnostic instrument assessing the degree of depression severity (33). Items asked about the frequency of experiencing problems related to the 9 DSM-IV criteria in the past 2 weeks, rated on a 4-point scale (0 = not at all to 3 = nearly every day). Items were summed to generate a total score of depressive symptoms (possible range of 0–27), with higher scores representing greater depression severity. Statistical reliability of the measure was high (Cronbach alpha=0.90). Because total PHQ-9 scores were skewed and not normally distributed, for analyses, responses were categorized by level of depression severity (minimal 0–4; mild 5–9; moderate 10–14; moderate severe 15–19; severe 20–27) according to the depression diagnostic status (33) and then dichotomized as low (minimal or mild 0–9) vs. high (moderate to severe 10–27) depression severity.

*Increase in depression severity:* Participants were also asked to rate the degree to which their feelings of depression have been bothering them now in comparison to before the pandemic on a 5-point Likert scale (1 = much less than before to 5 = much more than before). Responses were dichotomized as much less than before/a little less than before/the same as before vs. a little more than before/much more than before.

### 2.3. Analysis

We obtained descriptive statistics of sociodemographic characteristics (e.g., school grade, race, ethnicity, country of birth, gender), experiences and perceptions of anti-Asian violence, and depression severity for the study sample. Around 25% of our sample did not report their exact age in the survey. Therefore, we instead used participants' responses to their current school grade to infer their age; people who were in middle or high school were assumed to be adolescents below 18 years old, and those who were in college, in post-graduate school, or not currently in school were assumed to be young adults between the ages 18–29. We ran Fisher's exact tests to examine the bivariate relationships between sociodemographic characteristics, depression severity, and experiences and perceptions of anti-Asian violence. We used Fisher's exact tests to assess associations between our categorical variables because the test is valid for all sample sizes, whereas Chi-square tests of independence may be unreliable when the sample is small, particularly for scenarios of low expected cell frequencies (34, 35). For the bivariate analysis comparing depression severity and experiences/perceptions of anti-Asian violence by ethnicity, we included a subcategory of “mixed ethnicity” alongside subcategories for East Asian, Southeast Asian, South Asian ethnicity, since participants could select more than one ethnicity they identified as. Statistical significance was set to the *p* < 0.05 level.

To complement the quantitative results, we conducted thematic analysis (36) on the open-ended responses regarding their experiences with and feelings about anti-Asian violence and harassment during the pandemic. For each open-ended question, we reviewed all responses, developed codes to describe the content, identified patterns among the codes to create themes, and reviewed and refined the themes generated. We reported the most common theme for each open-ended question and included excerpts of written text from respondents that encapsulate the main theme in the results section. Coding was conducted in Microsoft Excel.

## 3. Results

Table 1 presents the univariate distribution of sociodemographic characteristics among our sample of 176 participants. Our sample comprised adolescents and young adults from 17 Asian ethnicities, with Chinese, Vietnamese, and Filipinx most represented (35, 27, and 18%, respectively), and from 35 states across the country, with half (49%) of participants from the western region of the US. Over two-thirds (69%) of our sample were cisgender women, with a minority (7%) being gender queer/nonbinary. Most participants (80%) identified their race as Asian only, while 20% indicated an additional race (e.g., American Indian/Alaska Native, Black, Hispanic, Native Hawaiian/Pacific Islander, or White). A fifth of respondents were born outside of the U.S. Most of our sample were young adults between the ages 18-29 (76%), and two-thirds of participants were currently in school: 15% were in middle or high school, 31% in college, and 11% in post graduate school.

**Table 1:** Distribution of sociodemographic characteristics among YAAHS respondents (*N* = 176).

	***n* (%)**
**Race**	
Single race Asian	141 (80.1)
Multiracial Asian	35 (19.9)
**Ethnicity** ^*^	
East Asian	84 (47.7)
Southeast Asian	100 (56.8)
South Asian	2 (1.1)
Not reported	3 (1.7)
**Age group** ^**^	
Adolescent (below 18 years old)	27 (15.3)
Young adult (18–29 years old)	134 (76.1)
Not reported	15 (8.5)
**School grade**	
Middle/high school	27 (15.3)
College	55 (31.2)
Post-graduate	20 (11.4)
Not currently in school	59 (33.5)
Not reported	15 (8.5)
**Gender**	
Cisgender man	26 (14.8)
Cisgender woman	120 (68.2)
Transgender man	2 (1.1)
Genderqueer/nonbinary	12 (6.8)
Not reported	16 (9.1)
**Country of birth**	
United States	127 (72.2)
Another county	35 (19.9)
Not reported	14 (7.9)
**US Region**	
Northeast	23 (13.1)
Midwest	13 (7.4)
South	30 (17.0)
West	87 (49.4)
Not reported	23 (13.1)

* Participants could report multiple ethnicities, so frequencies are calculated by dividing the n for each category by the total sample N (176) and do not add up to 100. East Asian includes Japanese, Korean, Chinese, Taiwanese, Tibetan, and Mongolian; Southeast Asian includes Vietnamese, Filipinx, Cambodian, Hmong, Malaysian, Thai, and Indonesian; and South Asian includes Indian, Bhutanese, Pakistani, and Sri Lankan.

** Age group was determined using participants' responses to school grade due to missing data on participants' exact ages.

**Figure 1:**
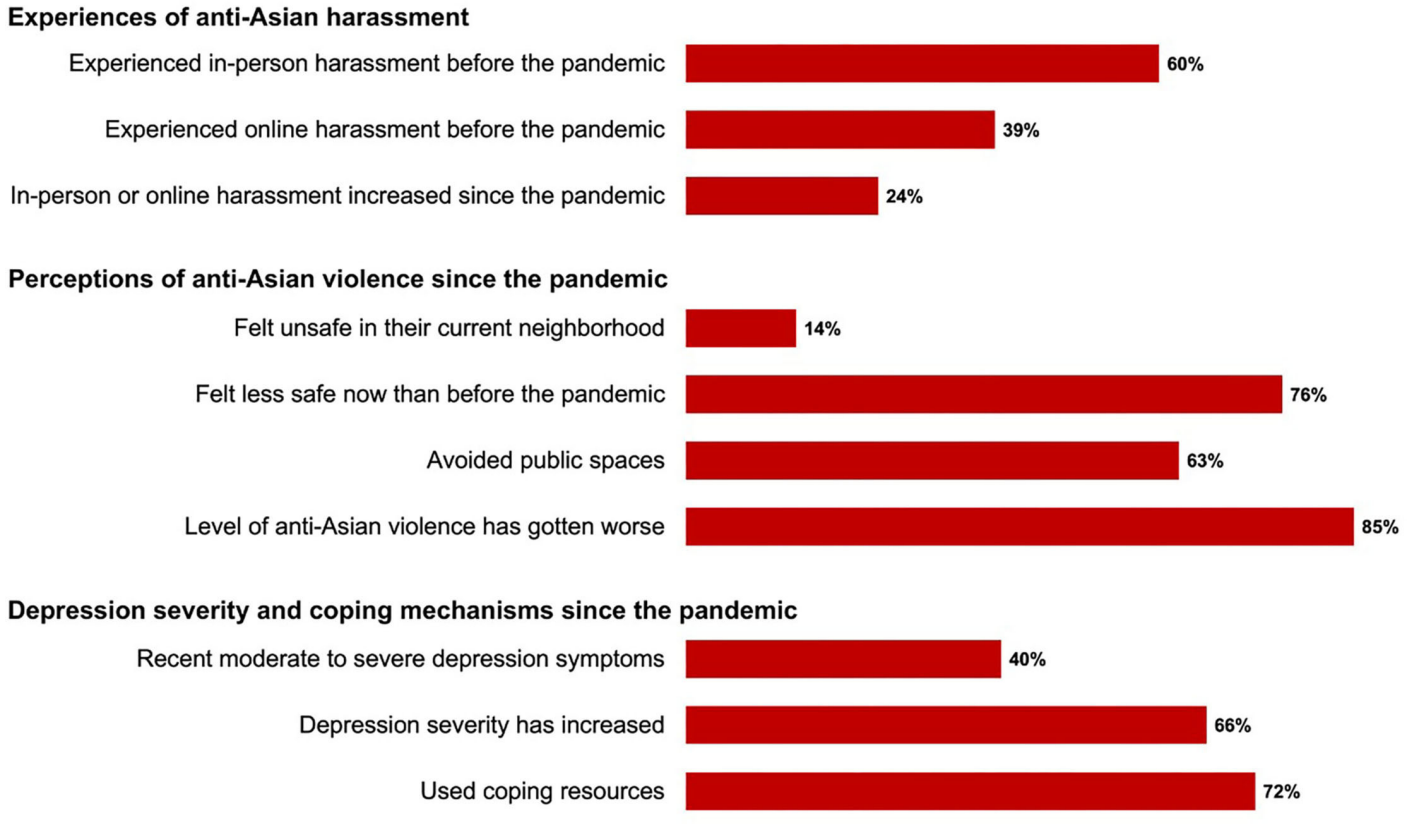
Distribution of perceptions and experiences of anti-Asian violence and depression severity since the pandemic among YAAHS respondents (*N* = 176).

Figure 1 displays the frequencies of experiences with anti-Asian harassment and depression severity before and since the COVID-19 pandemic started among our sample. Sixty percent of participants reported experiencing in-person, anti-Asian harassment before the pandemic and 39% reported experiencing online harassment. About a quarter (24%) of participants said that the frequency and/or severity of their personal experiences of in-person or online anti-Asian harassment has increased since the pandemic started.

Only 14% of participants indicated that they currently felt unsafe in their own neighborhood, but more than half (63%) reported avoiding public spaces since the pandemic started due to fear of being a target of anti-Asian violence. When asked to select from a list of what precautions they have taken to avoid anti-Asian violence, 75% of the sample reported that they avoided going to places alone, 64% avoided going places at night, 45% avoided specific neighborhoods or locations, and 23% begun carrying pepper-spray or mace.

In an open-ended response question about their changes in movement or behaviors in public spaces since the rise of anti-Asian violence during the pandemic, many participants stated that they do not go out as often as before the pandemic. If they do, many said that they try to be more cautious and vigilant of their surroundings and often feel uncomfortable and anxious in large, public spaces. For example, one participant said:

“I no longer ride the subway alone, and try to avoid using the subways at all if I can (I will walk, bike, or call an Uber if possible)... I purchased an annual Citibike membership in part to have an alternative method of transportation other than the subway.” (*23-year old Chinese American woman*)

Most participants reported feeling less safe now than before the pandemic (76%), and the majority (85%) indicated feeling like the level of anti-Asian violence had gotten worse since the pandemic started. When asked about how the level of anti-Asian violence has changed, many participants noted that although racism against Asians existed before the pandemic, the xenophobic rhetoric surrounding COVID-19 has amplified anti-Asian sentiment and increased the media coverage of anti-Asian incidents:

“Anti-Asian violence has always been present, yet our community didn't talk enough about it. Now, people are just blatantly being more violent and racist…it is undeniably true that after Trump's unacceptable name calling against the Asian community about COVID-19, that a rise in those crimes happened.” *(19-year old Korean American woman)*

“Hate crimes toward Asians have increased 8 fold. every time a major event like this occurs, america finds a scapegoat to thrust their violence onto…east and southeast Asians are taking the brunt of the violence because of covid.” *(20-year old Chinese American genderqueer/nonbinary person)*

In an open-response question asking participants how they felt when they saw or heard about hate crimes and racism toward Asian individuals since the pandemic started, the most common responses were feeling sad, angry, worried, frustrated, fearful, disappointed, anxious, scared, and hopeless. Additionally, several participants described feeling especially hurt and disturbed when seeing older Asian adults being targeted for hate crimes, leading them to be particularly concerned for the safety of their older family members and relatives. For example, a 17-year old Vietnamese man wrote that he felt “scared for my family, especially my grandparents and parents”.

Forty percent of our sample experienced moderate, moderately severe, or severe depressive symptoms in the past 2 weeks (19, 12, and 9%, respectively), and two-thirds indicated that their depressive symptoms have increased since the pandemic started. Most participants (72%) had engaged in some type of coping mechanism for dealing with the negative sentiment toward Asians since the pandemic began, including talking to their friends about it (84%), talking to their family (63%), reading about how to cope with it online (42%), going to therapy (24%), and participating in a support group online (12%).

Table 2 shows the sample distribution of those who experienced and perceived increased levels of anti-Asian violence since the pandemic and reported high depression severity by sociodemographic characteristic. A significantly larger proportion of participants who identified as mixed-race Asian reported an increase in experiences of anti-Asian harassment since the pandemic begun compared to those who identified as single-race Asian (37 vs. 21%, *p* = 0.04). Participants who were middle/high school (56%) or college students (56%) were significantly more likely to report high depression severity than participants who were in post-graduate school (25%) or not currently in school (26%) (*p* < 0.01). A significantly greater proportion of participants who were cisgender women (43%) or transgender/genderqueer (75%) had high depression severity compared to participants who were cisgender men (15%) (*p* < 0.01). Most notably, transgender/genderqueer (100%) and cisgender women (78%) participants were significantly more likely to feel less safe now than before the pandemic compared to cisgender men (65%). (*p* = 0.04).

**Table 2:** Distribution of sociodemographic characteristics by perceptions and experiences of anti-Asian violence and depression severity during the pandemic among YAAHS respondents (*N* = 176).

	**Felt less safe** **since the pandemic**			**In-person or online harassment increased** **since the pandemic**				**Recent depression severity**		
	**No**	**Yes**		**Never experienced harassment**	**No**	**Yes**		**Low**	**High**	
	**%**	**%**	***p-*** **value**	**%**	**%**	**%**	***p-*** **value**	**%**	**%**	***p-*** **value**
**Race**							^*^			
Single race Asian	21	79		37	42	21		59	41	
Multiracial Asian	27	74		17	46	37		59	41	
**Ethnicity**										
East Asian	24	76		34	39	27		59	41	
Southeast Asian	20	80		31	47	22		56	44	
South Asian	100	0		100	0	0		100	0	
Mixed ethnicity	15	85		31	38	31		69	31	
**Age group**										
Adolescent (below 18 years old)	19	82		30	33	37		44	56	
Young adult (18–29 years old)	23	78		34	45	22		62	38	
**School grade**										^**^
Middle/high school	19	81		30	33	37		44	56	
College	19	81		33	42	25		44	56	
Post-graduate	26	74		35	35	30		75	25	
Not currently in school	25	75		34	51	15		74	26	
**Gender**			^*^							^**^
Cis-gender man	35	65		27	54	19		85	15	
Cis-gender woman	22	78		36	42	23		57	43	
Transgender/Genderqueer/ Nonbinary	0	100		29	36	36		25	75	
**Country of birth**										
United States	20	80		31	44	25		61	39	
Another county	27	73		43	37	20		53	47	
**US region**										
Northeast	9	91		35	39	26		61	39	
Midwest	0	100		31	38	31		54	46	
South	21	79		33	47	20		68	32	
West	26	74		34	45	21		58	42	

Row percentages are reported. P-values are from results of Fisher's exact tests examining differences in sociodemographic characteristics by feelings of safety, experiences of harassment, and depression severity.

Significance codes:

** p < 0.01;

* p < 0.05.

**Figure 2:**
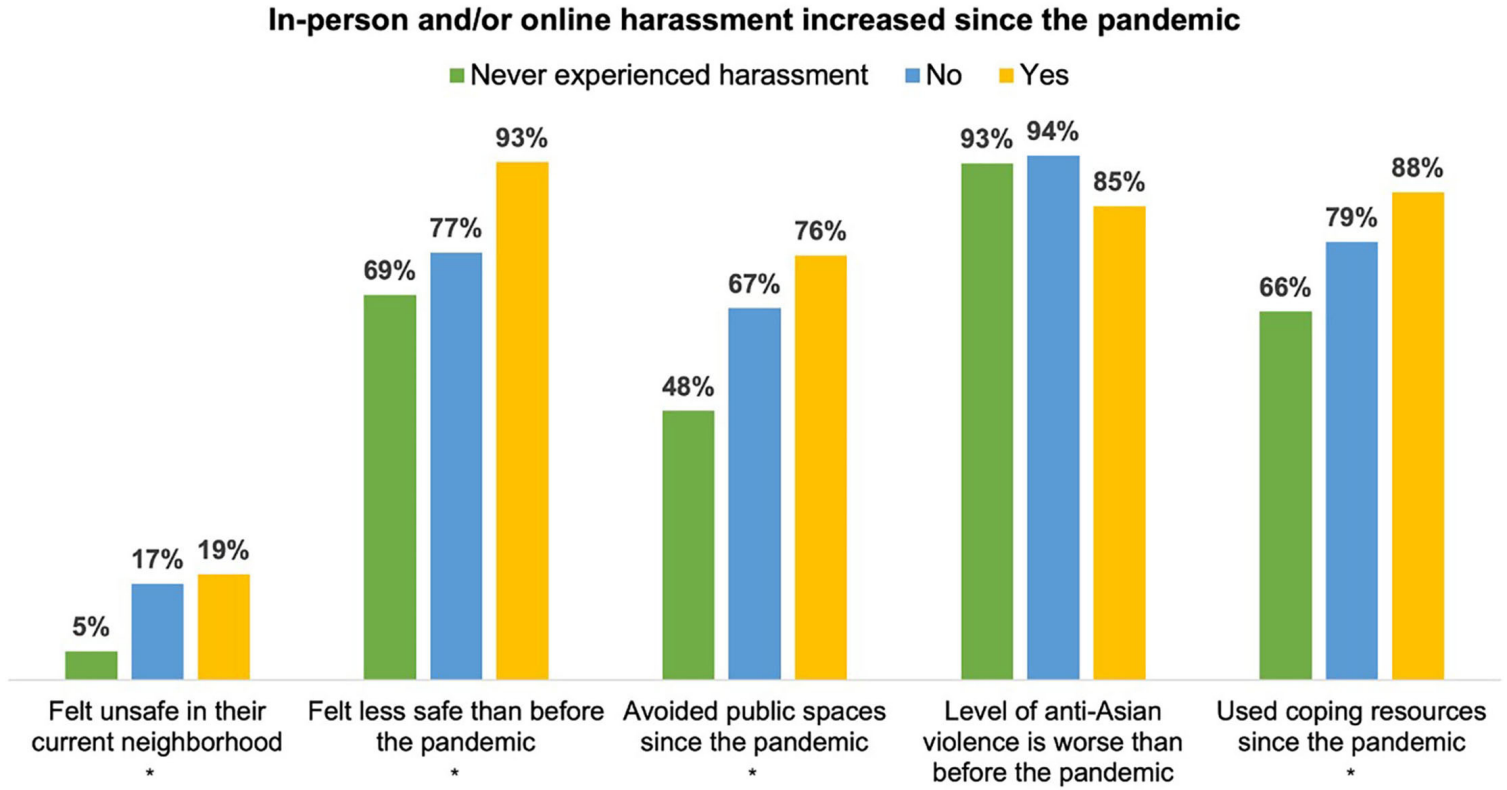
Distribution of perceptions of safety and of anti-Asian violence by personal experiences of harassment since the pandemic among YAAHS respondents (*N* = 176). *P-*values are from results of Fisher's exact tests examining differences in perceptions of safety and of anti-Asian violence by personal experiences of harassment. Significance codes: *p < 0.05.

Figure 2 displays the distribution of participants' perceptions of safety and anti-Asian violence by personal experiences of harassment following the pandemic. Participants who indicated experiencing increased levels of harassment since the pandemic were significantly more likely to indicate feeling unsafe in their current neighborhood (*p* = 0.05) and feeling less safe now than before the pandemic (*p* = 0.01) compared to those who did not experience increased harassment or never experienced harassment. These participants were also more likely to report avoiding public spaces due to fear of being a target of anti-Asian violence (*p* = 0.01) and using coping resources (*p* = 0.05) since the pandemic started than those who reported no increase in or never experienced harassment. These findings suggests that increases in personal, direct experiences of anti-Asian harassment during the pandemic contributed to decreased feelings of safety after the pandemic started.

Table 3 presents the distribution of depression severity by experiences and perceptions of safety and of anti-Asian violence since the pandemic. Recent high depression severity was not significantly associated with personal experiences of anti-Asian harassment before the pandemic or perceptions of safety since the pandemic began. However, participants were more likely to report that their depression symptoms had increased since the pandemic if they indicated feeling less safe now than before the pandemic (*p* < 0.01), avoiding public spaces due to fear of being a target of anti-Asian violence (*p* = 0.05), and feeling like the level of anti-Asian violence has gotten worse (*p* < 0.01).

**Table 3:** Distribution of perceptions of safety and anti-Asian violence since the pandemic by depression severity among YAAHS respondents (*N* = 176).

	**Recent depression severity**			**Depression severity increased** **since the pandemic**		
	**Low**	**High**	***p-*value**	**No**	**Yes**	***p-*value**
	**%**	**%**		**%**	**%**	
**Experienced in-person or online harassment**						
**before the pandemic**						
No	62	38		44	56	
Yes	57	43		29	71	
**In-person and/or online harassment increased**						
**since the pandemic**						
No	63	37		28	72	
Yes	49	51		33	67	
Never experienced harassment	61	39		43	57	
**Feelings of safety in their current neighborhood**						
Not safe	50	50		38	63	
Safe	60	40		34	66	
**Feelings of safety before and during the pandemic**						^**^
The same as before	69	31		62	38	
Less safe now than before	56	45		26	74	
**Avoided public spaces since the pandemic**						^*^
No	63	37		43	57	
Yes	57	44		28	72	
**Level of anti-Asian violence before and during the pandemic**						^**^
Same as before	64	36		71	29	
Worse than before	58	42		30	71	
**Used coping resources since the pandemic**						
No	49	51		46	54	
Yes	62	38		30	70	

Row percentages are reported. P-values are from results of Fisher's exact tests examining differences in depression severity by perceptions of safety and anti-Asian violence.

Significance codes:

* p < 0.05;

** p < 0.01.

## 4. Discussion

The Orientalist climate exacerbated by the COVID-19 pandemic has led to increased public attention toward anti-Asian racism. Prior to the pandemic, Asian American adolescents and young adults experienced varied levels and frequencies of racist harassment (37, 38) and depressive symptoms (15). Given the U.S. Surgeon General's declaration of the mental health crisis among adolescents and young adults and increased attention on anti-Asian violence, it is imperative that research and public policy must center on the mental health of Asian adolescents and young people (39).

Our study contributes to the emerging evidence base of anti-Asian violence experienced and felt by AA adolescents and young adults during the pandemic and how that is associated with depressive symptoms. These findings provide data for policymakers to invest in age- and culturally-appropriate care for this population. Additionally, study findings have implications on how best to measure depressive symptoms among AA adolescents and young adults. Below, we discuss our key findings.

Findings illustrate that most AA adolescents and young adults reported experiencing some form of in-person or online harassment prior to the pandemic. A quarter of the sample indicated that they experienced higher frequency and/or worse severity in harassment since the start of the pandemic. Among these young adults, they were more likely to report feeling less safe and avoiding public spaces due to fear of being a target of anti-Asian violence and having increased depressive symptoms. Additionally, transgender/genderqueer individuals and cisgender women were more likely to report high depression severity than cisgender men in our study.

We showed that participants who believed their depressive symptoms got worse during the pandemic compared to before were associated with several predictors: (1) they felt less safe now than prior to the pandemic, (2) avoided public spaces for fear of being a target of anti-Asian violence, and (3) felt like anti-Asian violence had gotten worse during the pandemic. These results contrasted with participants' recent depression severity scores, which measured a respondent's depressive symptoms within the past 2 weeks; those scores were not associated with anti-Asian harassment or perceptions of safety. One potential explanation for this nuanced finding could be that relative changes in perceptions of safety and perceptions of anti-Asian violence before and during the pandemic may not directly determine current mental health status among Asian/Asian American adolescents and young adults but may instead determine relative changes in mental health. Another possible reason could be attributed to the way in which our construct of mental health was operationalized and measured in this study. The PHQ-9 scale may not be sensitive enough to holistically capture the psychological impact of perceived discrimination and threats to safety during the pandemic. More specifically, PHQ-9 assesses clinical depression (33), but there are other aspects of adolescents' and young adults' mental wellbeing (i.e., anxiety, self-esteem, social connectedness) that could be affected by racial discrimination that we did not measure in this study (40–42). Future studies should consider a broader range of mental health outcomes to comprehensively explore the effects of anti-Asian racism during the pandemic on the wellbeing of adolescents and young adults who are racialized as Asians in the US.

Despite potential limitations in the mental health measure used in this study, our finding that increased perceptions of anti-Asian violence and threats to safety are related to perceived increases in depression severity during the pandemic support previous studies examining the negative and insidious impact of racial discrimination on mental health and adds to the burgeoning literature base on how the rise of anti-Asian racism during the COVID-19 pandemic has worsened the mental health of Asian Americans specifically (17, 43, 44). In addition to experiencing many of their formative schooling years online, due to pandemic safety precautions, Asian American adolescents and young adults' intentional avoidance of public spaces for fear of racial violence can come at the cost of sustaining social connection to support systems and having a sense of belonging in their neighborhoods, all of which may have long-term mental health consequences across the life course (45). Specifically, these experiences of isolation and anti-Asian racism may contribute to Asian American adolescents' developmental trajectory, which may be in more flux than young adults, such that it increases fear and mistrust of others, promoting intergroup hostility, concealing one's culture, and hinder positive identity development (12, 16–18).

Furthermore, our results on depression severity by gender, highlight and endorse the gendered disparities in mental health and experiences of violence (46). Cisgender girls and women, transgender, and genderqueer participants reported higher depression severity than cisgender boys and men. Scholarship in Asian American Studies and mental health show that the relationship between gendered racism and mental health is precipitated by labor, war, and migration histories of exotifying Asian women and femmes, subjecting them to misogynistic violence (47–51). Thus, future directions need to focus on the most marginalized groups in Asian American communities who are more likely to experience violence and depression.

### 4.1. Limitations

Our study conclusions should be considered in light of some limitations. The cross-sectional study design relies on retrospective self-report measures and thus may be subject to biased reporting, constricting our ability to draw causal conclusions or infer directionality. Our data are also unable to distinguish whether participants reported high recent depressive symptoms because of isolation during the COVID-19 pandemic or due to heightened anti-Asian violence. We were, however, able to establish a significant association between increased depression severity during the pandemic and feeling less safe now than before the pandemic because of increased anti-Asian violence. We used the adult version of PHQ-9 for all respondents regardless of age, and respondents ages 13–17 in our sample may have answered the questions on PHQ-9 differently if given the modified version of the instrument for adolescents. Additionally, our small sample size may have lacked adequate power to detect group differences. For instance, the extent to which our results highlighted gendered disparities in depression may be a reflection that over two-thirds of the sample identified as cisgender women and may be more open to recognizing/sharing depression symptoms on an online survey. The overrepresentation of young adults ages 18–29 in our survey may have biased our sample, so we caution against interpreting our study findings to adolescents younger than 18 years old. We also had a fairly large proportion of missing cases in our data due to unfinished questionnaires and were not able to thoroughly mitigate concerns for non-response bias, so findings should be interpreted considering potential selection bias. Furthermore, our convenience sampling design and utilization of a digital survey tool may have led to an over-representation of people who feel strongly about the survey topic and under-representation of people who are not digitally connected or who have restricted internet access, limiting our ability to generalize our study results to the U.S. AA adolescent and young adult population. Despite these limitations, we were able to conduct a rapid assessment of the impact of the rise of anti-Asian violence on the mental health and well-being of a sample of Asian American adolescents and young adults. Our findings support calls to conduct additional research examining the longer-term effects of anti-Asian violence on the health of AA adolescents and young adults and to implement targeted interventions to support their mental well-being.

## 5. Conclusion

YAAHS was launched following the racist and deadly Atlanta shootings of eight women, six of whom were Asian, by a White man (7). As a team of Asian American researchers, mostly women, femmes, and queer, it is not lost on us that anti-Asian racism and violence predated the COVID-19 pandemic. However, in the midst of a worsening Sinophobic climate, we sought to highlight the experiences of adolescents and young adults who are often left out of the picture. Asian American adolescents and young adults are experiencing multiple health and social crises stemming from anti-Asian racism, COVID-19, and fraught U.S. social safety nets. This requires an investment in violence prevention measures for both online and in-person settings from local and national public health and education officials. We insist officials, especially those in middle and high schools and colleges to encourage and promote a culture of reporting anti-Asian hate crimes and other hate incidents by promoting and investing in anti-hate campaigns and mental health services in public forums, and offering trainings focused on anti-racism and bystander intervention. Moreover, study results support the need for increased age- and culturally-appropriate mental healthcare and social support interventions for Asian American adolescents and young adults whose developmental trajectories may be affected. While our study did not specifically point to structural interventions, we urge policymakers from across sectors to invest in anti-carceral infrastructures of healing, safety, and support for those who are at risk for and impacted by anti-Asian hate and violence.

Lastly, we write directly to Asian American adolescents and young adults: we see you and the pain that you are going through. Rather than lean into social isolation, fear, and mistrust, we extend a warm invitation for you to engage in both self-care (e.g., accessing mental health services, limiting social media usage) and collective care. Regarding collective care, we urge you to find community and comfort with one another and with the many Asian American social justice groups around the country fighting for and with you.

## Data availability statement

The original contributions presented in the study are included in the article/supplementary material, further inquiries can be directed to the corresponding author/s.

## Ethics statement

The studies involving human participants were reviewed and approved by Population Council IRB. Written informed consent from the participants' legal guardian/next of kin was not required to participate in this study in accordance with the national legislation and the institutional requirements.

## Author contributions

JH, JC, AN, and TN contributed to the conception and design of the study. JC, AN, DH, and TD designed the questionnaire. EC, JC, and DH organized the dataset and uploaded it to Harvard Dataverse. JC led statistical analysis with support from JH. JH and JC led drafting of manuscript. AN wrote sections of the manuscript. All authors contributed to data collection. All authors contributed to the manuscript revision, read, and approved the submitted version.
